# Psychological Well-Being of Cancer Patients before and during the Pandemic: The Impact of COVID-19 Peritraumatic Distress

**DOI:** 10.3390/ijerph20054106

**Published:** 2023-02-25

**Authors:** Ilaria Bochicchio, Valentina Lucia La Rosa, Graziella Marino, Giuseppe Craparo, Elena Commodari, Giovanni Deiana, Francesca Sanseverino, Alfredo Tartarone, Raffaele Conca, Alessandro Rocco Lettini

**Affiliations:** 1 Unit of Clinical Psychology, Centro di Riferimento Oncologico della Basilicata (IRCCS-CROB), 85028 Rionero in Vulture, Italy; 2 Department of Educational Sciences, University of Catania, 95124 Catania, Italy; 3 Unit of Breast Surgery, Centro di Riferimento Oncologico della Basilicata (IRCCS-CROB), 85028 Rionero in Vulture, Italy; 4 Faculty of Human and Social Sciences, Kore University of Enna, 94100 Enna, Italy; 5 Unit of Oncological Gynecology, Centro di Riferimento Oncologico della Basilicata (IRCCS-CROB), 85028 Rionero in Vulture, Italy

**Keywords:** cancer, COVID-19, anxiety, depression, peritraumatic stress, quality of life

## Abstract

Background: This study aimed to evaluate the psychological impact of the COVID-19 pandemic on cancer patients. Methods: Ninety cancer patients undergoing chemotherapy with antiblastics were recruited from a tertiary medical center and completed a battery of standardized questionnaires to assess anxiety, depression, peritraumatic stress, and quality of life before and during the pandemic. Results: Quality of life worsened significantly during the pandemic compared with the pre-pandemic period. Anxiety and depression levels also increased significantly during the pandemic. COVID-19 peritraumatic distress significantly predicted lower quality-of-life scores during the pandemic. Conclusions: COVID-19 distress affected the overall quality of life of patients who already had lower levels of quality of life before the pandemic and who had advanced cancers. Cancer patients must receive adequate support from psychiatrists and psychologists to mitigate the psychological distress related to the pandemic.

## 1. Introduction

The World Health Organization declared COVID-19 a pandemic on 11 March 2020 [1]. To counter the spread of the virus and reduce the dramatic mortality rate of the disease, governments worldwide adopted restrictive social distancing and home isolation measures with several limitations to people’s personal and social lives [2,3], with detrimental effects on the mental health of the general population [3,4]. In fact, COVID-19 has been described as a stressful event that can cause the onset of psychopathological symptoms such as anxiety, depression, post-traumatic stress, sleep disorders, phobias, and fear of infection [5,6], especially in women and people at high risk of severe disease [3,7,8].

However, the literature has shown that some individuals are more at risk of going through negative psychological consequences related to the pandemic because they are at particularly critical stages of their life cycle. For example, adolescents and women in the postpartum period are recognized as some of the groups most vulnerable to the psychosocial impact of the pandemic [8,9].

The psychological effects of the pandemic may also be more significant for those who suffer from chronic diseases. In particular, cancer patients are particularly vulnerable to the psychological impact of the pandemic, as a cancer diagnosis is already associated with high psychological stress for patients and significant changes in their daily life, activities, and relationships, which pandemic-related distress may further exacerbate [10,11].

According to several studies, cancer patients are more susceptible to SARS-CoV-2 infection due to their more compromised immune defenses caused by treatment with immunosuppressive agents and chemotherapeutics [12]. In this regard, Lee et al. underlined a significantly higher risk of COVID-19 infection among cancer patients, especially older men [13]. Furthermore, for the same reasons, cancer patients are more likely to develop severe COVID-19, especially if they received chemotherapy in the months before the pandemic [12,14]. For these reasons, cancer patients tend to experience a more intense fear of contagion associated with a worse quality of life, especially for people who live alone and lack adequate social support [15].

Therefore, the COVID-19 pandemic may further exacerbate the psychological burden in cancer patients due to several factors: perception of a higher mortality risk [16], fear of contracting the virus [17], fear of an increased risk of complications if infected [16], fear of delays in receiving the necessary cancer therapies [18], and reduced social support from friends and family due to lockdown [19]. In this regard, it has been estimated that about half of cancer patients suffered from severe anxiety and mood disorders during the pandemic, while 30% manifest mild forms [20].

Furthermore, due to pandemic-related restrictions, people with cancer had to adhere to social distancing to a greater extent than others [4,21] and faced the closure of support centers, foundations, and associations where they could share their experience with other people with the same pathology [22]. This condition undoubtedly contributed to the increased feelings of anxiety, depression, and loneliness that affected the quality of life of these patients [17,23].

It should also be considered that cancer patients have often faced suspension or postponement of cancer follow-ups and treatments [18]. The COVID-19 pandemic had a considerable impact on the organization of healthcare systems around the world. In fact, beds and staff have been reconverted and assigned to emergency management of patients with COVID [24,25]. Additionally, routine clinical care and elective treatments (including antitumor therapies) were temporarily suspended [26]. Indeed, this factor also significantly increased the risk of adverse psychological consequences for cancer patients during the pandemic [17,18,21].

There is ample evidence that psychological distress and quality of life significantly affect the course and outcomes of cancer treatment [18]. Compared with patients without depression, patients with depression are more likely to be non-compliant with their medical treatment, which increases mortality rates [27,28]. Consequently, it is essential to investigate the psychological impact of the COVID-19 pandemic on cancer patients, as it is a critically important variable for disease progression and treatment adherence.

In light of these considerations, this study evaluated anxiety, depression, peritraumatic stress, and quality of life before and during the pandemic in a sample of patients treated in the Department of Oncology and Surgery of IRCCS-CROB in Rionero in Vulture (Italy).

Specifically, our research hypothesis was that cancer patients reported higher levels of anxiety and depression, and lower quality of life during the pandemic than during the pre-pandemic period, and that COVID-19 peritraumatic distress had a significant impact on these variables.

## 2. Materials and Methods

### 2.1. Study Design and Participants

This observational study was carried out at the Department of Oncology and Surgery of the IRCCS-CROB in Rionero in Vulture (Italy) and enrolled patients with different types and stages of cancer undergoing antiblastic chemotherapy. Patients with a history of ongoing psychological care and/or psychiatric treatment (not including psychological counselling related to cancer) were excluded from the sample.

Participants were evaluated in two phases (T0 and T1). T0 covered the pre-pandemic period between January and February 2020. T1 occurred a year later, during the second lockdown in Italy.

At T0, a recruitment interview was conducted to collect clinical and anamnestic data. The patients then completed a battery of standardized questionnaires to assess anxiety, depression, and quality of life. At T1, COVID-19 peritraumatic distress was evaluated along with the variables already investigated at the beginning of the study.

Informed consent was obtained from all participants after explaining the objectives and the study design. The study protocol was drafted according to the Declaration of Helsinki standards and approved by the Ethics Committee of IRCCS-CROB (protocol no. 1497, 19 April 2022).

### 2.2. Setting

At T1, a series of restrictions were in place at the center where the study was conducted to contain the spread of COVID-19. Specifically, access to the ward was allowed only to the patient without companions, except in cases of severe difficulties in the patient’s autonomy. Similarly, access to the Day Hospital was granted only while wearing protective equipment (mask) and without attendants. Patients to be admitted were swabbed in a dedicated space within the ward and were admitted only if the result was negative. Furthermore, the inpatient could be assisted only in the case of severe difficulties in autonomy. In this case, the caregiver was also swabbed and admitted to the ward only if the result was negative. Visits to hospitalized patients were in no way allowed. Finally, healthcare personnel were to be mandatorily vaccinated against COVID-19, and access to the hospital was allowed only to those with a Green Pass (a certificate required in Italy attesting COVID-19 vaccination or recovery from disease or a negative COVID test).

### 2.3. EORTC Quality of Life Questionnaire Version 3.0 (EORTC QLQ-C30)

The Italian version of the EORTC QLQ-C30 [29,30] was used to assess the patients’ quality of life. It is a 30-item questionnaire (e.g., “Do you need to stay in bed or a chair during the day?”, “Were you limited in pursuing your hobbies or other leisure time activities?”, “Has your physical condition or medical treatment interfered with your family life?”) consisting of five functional scales (physical functioning, social functioning, role functioning, emotional functioning, cognitive functioning), nine symptom scales (fatigue, pain, nausea/vomiting, dyspnea, sleep disturbances, appetite loss, diarrhea, constipation, and financial difficulties), and a global health/quality-of-life scale. The raw scores were transformed into scale scores ranging from 0 to 100. Higher scores on the functional and global health scales indicate a better quality of life, while higher scores on the symptomatic scales indicate a high level of symptoms. The Italian version of the EORTC QLQ-C30 has been validated with good psychometric properties (Cronbach’s α greater than 0.70) [31].

### 2.4. Hospital Anxiety and Depression Scale (HADS)

The Italian version of the HADS consists of 14 items and is primarily used to assess anxiety and depression in cancer patients [32,33]. It includes seven items that consider cognitive and emotional aspects of depression and seven that focus on anxiety symptoms. Each item (e.g., “I get a sort of frightened feeling as if something awful is about to happen”, “I have lost interest in my appearance”) is scored on a four-point Likert scale ranging from 0 (*Not at all*) to 3 (*Most of the time*). It is possible to calculate the scores of two subscales: the anxiety subscale (HADS-A) and the depression subscale (HADS-D). The Italian translation of the questionnaire was already used in clinical samples with good psychometric properties (Cronbach’s α between 0.80 and 0.85) [32,34].

### 2.5. COVID-19 Peritraumatic Distress Index (CPDI)

The COVID-19 Peritraumatic Distress Index (CPDI) was used to assess peritraumatic distress during the pandemic [35,36]. The questionnaire comprises 24 items (e.g., “Compared to usual, I feel more nervous and anxious”, “I collect information about COVID-19 all day. Even if it is not necessary, I cannot stop myself”, “I cannot sleep well. I always dream about myself or my family being infected by COVID-19”) rated on a 5-point scale ranging from 0 (*Not at all*) to 4 (*extremely*).

The total score ranges from 0 to 100. A score below 28 indicates normal distress, between 28 and 51 indicates mild-to-moderate distress, and above 51 indicates severe distress. The Italian version of the CPDI showed good psychometric properties (Cronbach’s α = 0.92) [36].

### 2.6. Statistical Analyses

Firstly, we screened the dataset for missing data and examined the normality of the variables. Then, we performed a post hoc sensitivity power analysis to determine the strength of the effects that can be reliably detected in this study.

Mean (M) ± standard deviation (SD) was used for continuous variables, while categorical variables were expressed as frequencies and percentages.

A one-way repeated-measures multivariate analysis of variance (MANOVA) with EORTC QLQ-C30 and HADS scores as the dependent variables and time (before and during the COVID-19 pandemic) as the within-subject factor was used to investigate the differences in patients’ quality of life, anxiety, and depression during the pandemic compared with the pre-pandemic period. In addition, a one-way MANOVA with EORTC QLQ-C30 and HADS scores as the dependent variables and CPDI scores (normal vs mild–moderate distress) as the between-subject factor was performed to explore the impact of COVID-19 peritraumatic distress levels on patients’ quality of life and psychological well-being.

Finally, a multiple regression model was developed to identify the main predictors of quality-of-life scores in cancer patients during the pandemic. Sociodemographic and clinical variables, quality-of-life scores at baseline, COVID-19 peritraumatic distress, and anxiety and depression scores were used as independent variables, while the quality-of-life scores during the pandemic were the dependent variable.

## 3. Results

### 3.1. Sample

No missing data were found during the preliminary analysis. The total sample consisted of 90 patients. Overall, 76.7% were women, with a mean age of 56.2 years (*SD* = 11.4, range: 35–83). Most of the sample had urogenital or breast cancer (71.1%). In the sample, 35.6% had stage IV cancer, while only 5.6% had cancer recurrence. In addition, 37.8% of the sample had at least one other medical condition besides cancer. Finally, only 14.4% of the sample was affected by COVID-19. Table 1 reports all the characteristics of the patients.

### 3.2. Sensitivity Power Analysis

Considering our primary aim, with a sample size of 90 individuals, a power of 0.80, 2 groups, 8 response variables, and α  =  0.05, the present sample size was adequate to detect a minimum effect of f^2^ = 0.18, which is considered a medium effect [37]. The analysis was performed with G*Power 3.1 [38].

### 3.3. Differences in Quality-of-Life, Anxiety, and Depression Scores before and during the COVID-19 Pandemic

A one-way repeated-measures MANOVA (independent variable = time) with eight dependent variables (factors = global health status/QoL, physical functioning, role functioning, emotional functioning, cognitive functioning, social functioning, anxiety, and depression) was run. There were no significant violations of normality, linearity, univariate or multivariate outliers, homogeneity of variance–covariance matrices, or multicollinearity observed during preliminary assumption testing.

Significant differences were found in the EORTC-QLQ-C30 and HADS scores before and during the COVID-19 pandemic (Wilks’s Λ = 0.295, F(8, 82) = 24.50, *p* < 0.001). Specifically, follow-up analyses of variance (ANOVAs) on each dependent variable showed significant differences between T0 and T1 on global health status (*p* < 0.001), physical functioning (*p* < 0.001), role functioning (*p* < 0.001), emotional functioning (*p* < 0.001), cognitive functioning (*p* = 0.001), anxiety (*p* < 0.001), and depression (*p* < 0.001).

As shown in Table 2, the global quality of life decreased from an average of 65.77 ± 15.20 to 62.51 ± 13.23 on the EORTC QLQ-C30 scale. Regarding the EORTC QLQ-C30 functioning scales, we observed a significant decrease in scores from baseline to follow-up in physical functioning (from 75.64 ± 17.23 to 70.78 ± 15.38), role functioning (from 73.19 ± 24.04 to 70.89 ± 24.13), emotional functioning (from 77.04 ± 19.55 to 74.09 ± 19.69), and cognitive functioning (from 84.18 ± 15.52 to 82.57 ± 16.88). Depression scores increased from baseline to follow-up, from 4.04 ± 2.05 before the pandemic to 5.96 ± 2.79 during the pandemic. Finally, anxiety symptoms also increased significantly, from a score of 5.31 ± 2.62 before the pandemic to 7.31 ± 2.88 during the pandemic.

### 3.4. Differences in Quality-of-Life, Anxiety, and Depression Scores Based on the COVID-19 Distress Levels

Based on the CPDI scores of the sample, it was possible to divide the study sample into two groups: patients with normal COVID-19 distress levels (scores < 28) (*n* = 56) and patients with mild–moderate COVID-19 distress levels (scores between 28 and 51) (*n* = 34). No patients reported severe levels of COVID-19 distress (scores > 51).

As a second step of our analysis, a one-way MANOVA (independent variable = COVID-19 distress levels) with eight dependent variables (factors = global health status/QoL, physical functioning, role functioning, emotional functioning, cognitive functioning, social functioning, anxiety, and depression) was conducted to explore the effects of COVID-19 peritraumatic distress levels on patients’ quality of life, anxiety, and depression. There were no significant violations of normality, linearity, univariate or multivariate outliers, homogeneity of variance–covariance matrices, or multicollinearity observed during preliminary assumption testing.

Significant differences were found in the EORTC-QLQ-C30 and HADS scores based on the levels of COVID-19 distress (Wilks’s Λ = 0.785, F(8, 81) = 2.77, *p* = 0.009). However, follow-up analyses of variance (ANOVAs) on each dependent variable showed significant differences only in anxiety (*p* < 0.001) and depression (*p* < 0.001).

As shown in Table 3, patients with mild–moderate levels of COVID-19 distress reported significantly higher scores of anxiety (8.62 ± 2.69) and depression (6.85 ± 2.87) than patients with normal levels of COVID-19 distress (6.52 ± 2.71 and 5.41 ± 2.62).

### 3.5. Predictors of Quality-of-Life Scores during the Pandemic

As a final step, we conducted a multiple linear regression analysis to assess the main predictors of global quality of life in cancer patients during the pandemic.

All regression assumptions were checked. Partial regression plots and a plot of studentized residuals against predicted values showed linearity. A Durbin–Watson value between 1.5 and 2.5 (2.1) showed independence of residuals. Visual inspection of the plot of studentized residuals versus unstandardized predicted values indicated homoscedasticity. There was no evidence of multicollinearity, as assessed by tolerance values greater than 0.1. There were no studentized deleted residuals greater than ±3 standard deviations, no leverage values greater than 0.2, or values for Cook’s distance above 1. Based on a Q–Q plot, the assumption of normality was met.

The regression model explained 92% of the variance (R^2^ = 0.92, F = 60.19, *p* < 0.001). Specifically, the independent variables that contribute the most to explaining the quality-of-life scores in T1 are quality of life at baseline (β = 0.95, *p* < 0.001), cancer stage (β = −0.09, *p* = 0.02), and peritraumatic COVID-19 distress (β = −0.04, *p* = 0.03). Table 4 shows all the results of the regression analysis and the contribution of the other independent variables.

## 4. Discussion

This study aimed to explore and compare anxiety, depression, peritraumatic distress, and quality of life before and during the pandemic in a sample of cancer patients. Furthermore, we investigated the impact of COVID-19 peritraumatic distress on patients’ general quality of life and psychological well-being during the pandemic.

As hypothesized, our findings showed that there was a significant decline in quality of life during the period of the pandemic as compared to the pre-pandemic period. In particular, cancer patients in our sample reported significantly lower scores for physical, role, emotional, and cognitive functioning during the pandemic than at baseline. On the contrary, we did not find significant changes in social functioning, probably because people with cancer already take more infection prevention measures than the general population [39], so it is likely that social distancing measures did not particularly affect social functioning and global quality of life of patients in our sample.

Interestingly, the quality-of-life scores of our sample both before and during the pandemic were in line with the normative baseline values of the questionnaire, although they worsened during the pandemic period. This finding can be explained by the fact that our sample consists entirely of cancer patients who were diagnosed before the pandemic outbreak and had already started chemotherapy treatment. In fact, it has been underlined that patients who started chemotherapy or radiotherapy before the pandemic entered a phase of adaptation that helps them regain control. In this phase, they could handle stress and apply resilience mechanisms, that is, adaptation to the situation [40].

According to data already reported in other studies, anxiety and depression levels also increased significantly during the pandemic [23,39,41]. In this sense, cancer patients already suffer a heavy psychological burden due to their disease and treatments. However, the pandemic undoubtedly contributed to exacerbating this condition of psychological distress. In fact, patients in our sample who reported mild-to-moderate levels of COVID-19 peritraumatic distress had higher levels of anxiety and depression than patients with normal stress levels. Therefore, our findings confirm that COVID-19 represented a highly stressful event for cancer patients [17,23,39,41], especially in relation to emotional components such as anxiety and depression.

Further confirming these data, COVID-19 peritraumatic distress was found to be a significant predictor of lower overall quality-of-life scores in our sample. Cancer stage also significantly predicts quality-of-life scores. In particular, patients with more advanced cancer stage report lower quality-of-life scores, as already reported in the literature [23,39,40]. However, the variable in our model that most strongly explains the variation in the quality-of-life scores is the quality-of-life score at baseline. In other words, patients with good quality-of-life levels before the pandemic will report higher scores than those with lower scores at baseline. According to these data, the pandemic is undoubtedly a factor that may contribute to the psychological distress of cancer patients, but not in an absolute sense. In fact, pandemic-related distress affected more the overall quality of life of those patients who already had lower levels of quality of life before the pandemic and who had advanced cancers. As a result, these patients need more support, as they are more at risk of suffering the emotional impact of the pandemic. This finding is also in line with studies conducted during the pandemic on various groups most at risk of experiencing negative psychological consequences (i.e., adolescents or pregnant and postpartum women) which have shown that the traumatic impact of the pandemic depends on a combination of risk and protective factors that can mitigate or exacerbate the psychosocial effects of the event [5,6,9,42,43].

Our study has limitations that must be taken into account. First, the sample size is relatively small, although the sensitivity analysis showed our sample’s suitability to detect a medium effect. Second, we reported the experience of a single cancer center and a single geographical region in Italy. Therefore, conducting studies involving different centers in different geographical areas and with larger samples would be appropriate to compare the results obtained. In addition, we only included patients undergoing antiblastic chemotherapy in our sample, so further studies should also consider the type of cancer treatment to evaluate any differences in quality of life and psychological well-being between patients undergoing different types of treatment. Another limitation of this study is that our center never suspended cancer treatments during the pandemic, so patients did not experience anxiety about the delay or suspension of their cancer treatments, which may affect their overall quality of life. This should certainly be considered when evaluating our study’s results and comparing them with those of other studies in the literature.

Additionally, there was no control group whose results could be compared with those of the sample of cancer patients that we analyzed. Therefore, future studies should also include a group of healthy subjects to improve further the significance of the results obtained.

## 5. Conclusions

Our study shows the significant impact of the COVID-19 pandemic on the quality of life and emotional well-being of patients with cancer undergoing antiblastic chemotherapy. There is a need for further studies to identify risk and protective factors related to the psychological effects of the pandemic in this specific group of people.

More specifically, it is particularly important for cancer patients to receive adequate support from psychiatrists and psychologists during the pandemic to alleviate the psychological distress related to this unpredictable event. Additionally, it is crucial for doctors, cancer care professionals, as well as cancer patients to improve communication, since effective communication can improve patients’ ability to adhere to treatments and their general quality of life, even during the COVID-19 pandemic.

## Figures and Tables

**Table 1:** Characteristics of the sample.

Variable		Value
Gender	Female (%)	69 (76.7)
	Male (%)	21 (23.3)
Age	Mean ± *SD*	56.2 ± 11.4
Years of education	Mean ± *SD*	12.3 ± 3.8
Cancer type	Hematologic (%)	7 (7.8)
	Gastrointestinal (%)	9 (10.0)
	Lung (%)	7 (7.8)
	Urogenital and breast (%)	64 (71.1)
	Endocrine (%)	3 (3.3)
Cancer stage	I (%)	28 (31.1)
	II (%)	23 (25.6)
	III (%)	7 (7.8)
	IV (%)	32 (35.6)
Cancer recurrence	Yes (%)	5 (5.6)
	No (%)	85 (94.4)
Number of comorbidities	0 (%)	56 (62.2)
	1 (%)	17 (18.9)
	2 (%)	14 (15.6)
	>2 (%)	3 (3.3)
Affected by COVID-19	Yes (%)	13 (14.4)
	No (%)	77 (85.6)

**Table 2:** Means and standard deviations of EORTC-QLQ-C30 and HADS scores before and during the COVID-19 pandemic.

Scale	Pre-COVID-19	During COVID-19
**EORTC-QLQ-C30**		
Global health status/QoL	65.77 (15.20)	62.51 (13.23)
*Functional scales*		
Physical functioning	75.64 (17.23)	70.78 (15.38)
Role functioning	73.19 (24.04)	70.89 (24.13)
Emotional functioning	77.04 (19.55)	74.09 (19.69)
Cognitive functioning	84.18 (15.52)	82.57 (16.88)
Social functioning	82.26 (19.84)	82.57 (16.88)
**HADS**		
Anxiety	5.31 (2.62)	7.31 (2.88)
Depression	4.04 (2.05)	5.96 (2.79)

*Note*. Standard deviations are presented in parentheses. EORTC-QLQ-C30 = European Organisation for Research and Treatment of Cancer core questionnaire; HADS = Hospital Anxiety and Depression Scale.

**Table 3:** Means and standard deviations of EORTC-QLQ-C30 and HADS scores according to COVID-19 stress levels.

Scale	Normal COVID-19 Stress (*n* = 56)	Mild-Moderate COVID-19 Stress (*n* =34)
**EORTC-QLQ-C30**		
Global health status/QoL	63.79 (12.56)	60.41 (14.19)
*Functional scales*		
Physical functioning	73.24 (12.14)	69.29 (16.99)
Role functioning	74.70 (20.92)	64.62 (27.85)
Emotional functioning	76.00 (19.04)	70.94 (20.60)
Cognitive functioning	83.05 (17.72)	81.76 (15.62)
Social functioning	82.68 (18.14)	80.44 (20.94)
**HADS**		
Anxiety	6.52 (2.71)	8.62 (2.69)
Depression	5.41 (2.62)	6.85 (2.87)

*Note*. Standard deviations are presented in parentheses. EORTC-QLQ-C30 = European Organisation for Research and Treatment of Cancer core questionnaire; HADS = Hospital Anxiety and Depression Scale.

**Table 4:** Predictors of quality-of-life scores in cancer patients during the pandemic.

	*β*	*t*	*p*
(Intercept)		2.67	0.01
Gender	−0.06	−1.30	0.20
Age	−0.01	−0.16	0.88
Years of education	−0.01	−0.25	0.80
*Cancer type*			
Hematologic	0	0	
Gastrointestinal	0.04	0.70	0.48
Lung	0.06	1.06	0.29
Urogenital	−0.02	−0.28	0.78
Endocrine	0.01	0.12	0.90
Cancer stage	−0.09	−2.46	0.02
Number of comorbidities	0.02	0.64	0.53
Affected by COVID-19	0.05	1.41	0.16
CPDI	−0.04	−0.85	0.03
Quality of life at baseline	0.95	27.41	0.00
HADS Anxiety	−0.03	−0.82	0.41
HADS Depression	−0.01	−0.22	0.82
R^2^	0.92		

Note: *n* = 90. HADS = Hospital Anxiety and Depression Scale; CPDI = COVID-19 Peritraumatic Distress Index.

## Data Availability

The data presented in this study are available on request from the corresponding author. The data are not publicly available to ensure the privacy of the patients participating in the study.

## References

[1] World Health Organization WHO Announces COVID-19 Outbreak a Pandemic. https://www.euro.who.int/en/health-topics/health-emergencies/coronavirus-covid-19/news/news/2020/3/who-announces-covid-19-outbreak-a-pandemic.

[2] Rana U., Govender J. (2022). Exploring the Consequences of the COVID-19 Pandemic: Social, Cultural, Economic, and Psychological Insights and Perspectives.

[3] Passavanti M., Argentieri A., Barbieri D.M., Lou B., Wijayarastna K., Foroutan Mirhosseini A.S., Wang F., Naseri S., Qamhia I., Tangeras M. (2021). The psychological impact of COVID-19 and restrictive measures in the world. J. Affect. Disord..

[4] Brooks S.K., Webster R.K., Smith L.E., Woodland L., Wessely S., Greenberg N., Rubin G.J. (2020). The psychological impact of quarantine and how to reduce it: Rapid review of the evidence. Lancet.

[5] Craparo G., La Rosa V.L., Marino G., Vezzoli M., Cina G.S., Colombi M., Arcoleo G., Severino M., Costanzo G., Mangiapane E. (2022). Risk of post-traumatic stress symptoms in hospitalized and non-hospitalized COVID-19 recovered patients. A cross-sectional study. Psychiatry Res..

[6] La Rosa V.L., Gori A., Faraci P., Vicario C.M., Craparo G. (2021). Traumatic Distress, Alexithymia, Dissociation, and Risk of Addiction During the First Wave of COVID-19 in Italy: Results from a Cross-sectional Online Survey on a Non-clinical Adult Sample. Int. J. Ment. Health Addict..

[7] Xiong J., Lipsitz O., Nasri F., Lui L.M.W., Gill H., Phan L., Chen-Li D., Iacobucci M., Ho R., Majeed A. (2020). Impact of COVID-19 pandemic on mental health in the general population: A systematic review. J. Affect. Disord..

[8] Oddo-Sommerfeld S., Schermelleh-Engel K., Konopka M., La Rosa V.L., Louwen F., Sommerlad S. (2022). Giving birth alone due to COVID-19-related hospital restrictions compared to accompanied birth: Psychological distress in women with caesarean section or vaginal birth—A cross-sectional study. J. Perinat. Med..

[9] Commodari E., La Rosa V.L. (2020). Adolescents in Quarantine During COVID-19 Pandemic in Italy: Perceived Health Risk, Beliefs, Psychological Experiences and Expectations for the Future. Front. Psychol..

[10] Grajek M., Krupa-Kotara K., Rozmiarek M., Sobczyk K., Dzialach E., Gorski M., Kobza J. (2022). The Level of COVID-19 Anxiety among Oncology Patients in Poland. Int. J. Environ. Res. Public Health.

[11] Salamanca-Balen N., Qiu M., Merluzzi T.V. (2021). COVID-19 pandemic stress, tolerance of uncertainty and well-being for persons with and without cancer. Psychol. Health.

[12] Liang W., Guan W., Chen R., Wang W., Li J., Xu K., Li C., Ai Q., Lu W., Liang H. (2020). Cancer patients in SARS-CoV-2 infection: A nationwide analysis in China. Lancet Oncol..

[13] Lee K.A., Ma W., Sikavi D.R., Drew D.A., Nguyen L.H., Bowyer R.C.E., Cardoso M.J., Fall T., Freidin M.B., Gomez M. (2021). Cancer and Risk of COVID-19 Through a General Community Survey. Oncologist.

[14] Miyashita H., Mikami T., Chopra N., Yamada T., Chernyavsky S., Rizk D., Cruz C. (2020). Do patients with cancer have a poorer prognosis of COVID-19? An experience in New York City. Ann. Oncol..

[15] Ciazynska M., Pabianek M., Szczepaniak K., Ulanska M., Skibinska M., Owczarek W., Narbutt J., Lesiak A. (2020). Quality of life of cancer patients during coronavirus disease (COVID-19) pandemic. Psychooncology.

[16] Islam J.Y., Vidot D.C., Camacho-Rivera M. (2021). Evaluating Mental Health–Related Symptoms Among Cancer Survivors During the COVID-19 Pandemic: An Analysis of the COVID Impact Survey. JCO Oncol. Pract..

[17] Jeppesen S.S., Bentsen K.K., Jorgensen T.L., Holm H.S., Holst-Christensen L., Tarpgaard L.S., Dahlrot R.H., Eckhoff L. (2021). Quality of life in patients with cancer during the COVID-19 pandemic—A Danish cross-sectional study (COPICADS). Acta Oncol..

[18] Ye Y., Wang J., Cai S., Fu X., Ji Y. (2022). Psychological distress of cancer patients caused by treatment delay during the COVID-19 pandemic in China: A cross-sectional study. Psychooncology.

[19] Moraliyage H., De Silva D., Ranasinghe W., Adikari A., Alahakoon D., Prasad R., Lawrentschuk N., Bolton D. (2021). Cancer in Lockdown: Impact of the COVID-19 Pandemic on Patients with Cancer. Oncologist.

[20] Marroquín B., Vine V., Morgan R. (2020). Mental health during the COVID-19 pandemic: Effects of stay-at-home policies, social distancing behavior, and social resources. Psychiatry Res..

[21] van de Haar J., Hoes L.R., Coles C.E., Seamon K., Frohling S., Jager D., Valenza F., de Braud F., De Petris L., Bergh J. (2020). Caring for patients with cancer in the COVID-19 era. Nat. Med..

[22] Reizer A., Geffen L., Koslowsky M. (2021). Life under the COVID-19 lockdown: On the relationship between intolerance of uncertainty and psychological distress. Psychol. Trauma Theory Res. Pract. Policy.

[23] Yildirim O.A., Poyraz K., Erdur E. (2021). Depression and anxiety in cancer patients before and during the SARS-CoV-2 pandemic: Association with treatment delays. Qual. Life Res..

[24] Lewis P.J., Roques T.W. (2020). The Response of the UK Clinical Oncology Community to the COVID-19 Pandemic. Clin. Oncol..

[25] Santin O., Mc Mullan J., Jenkins C., Anderson L.A., Mc Shane C.M. (2022). Supporting someone with cancer during the COVID-19 pandemic: A mixed methods analysis of cancer carer’s health, Quality of Life and need for support. Health Soc. Care Community.

[26] Bakouny Z., Hawley J.E., Choueiri T.K., Peters S., Rini B.I., Warner J.L., Painter C.A. (2020). COVID-19 and Cancer: Current Challenges and Perspectives. Cancer Cell.

[27] Pinquart M., Duberstein P.R. (2010). Depression and cancer mortality: A meta-analysis. Psychol. Med..

[28] DiMatteo M.R., Lepper H.S., Croghan T.W. (2000). Depression Is a Risk Factor for Noncompliance With Medical Treatment. Arch. Intern. Med..

[29] AAaronson N.K., Ahmedzai S., Bergman B., Bullinger M., Cull A., Duez N.J., Filiberti A., Flechtner H., Fleishman S.B., De Haes J.C.J.M. (1993). The European Organization for Research and Treatment of Cancer QLQ-C30: A Quality-of-Life Instrument for Use in International Clinical Trials in Oncology. JNCI J. Natl. Cancer Inst..

[30] Pilz M.J., Gamper E.-M., Efficace F., Arraras J.I., Nolte S., Liegl G., Rose M., Giesinger J.M. (2022). EORTC QLQ-C30 general population normative data for Italy by sex, age and health condition: An analysis of 1036 individuals. BMC Public Health.

[31] Apolone G., Filiberti A., Cifani S., Ruggiata R., Mosconi P. (1998). Evaluation of the EORTC QLQ-C30 questionnaire: A comparison with SF-36 Health Survey in a cohort of Italian long-survival cancer patients. Ann. Oncol..

[32] Costantini M., Musso M., Viterbori P., Bonci F., Del Mastro L., Garrone O., Venturini M., Morasso G. (1999). Detecting psychological distress in cancer patients: Validity of the Italian version of the Hospital Anxiety and Depression Scale. Support. Care Cancer.

[33] Annunziata M.A., Muzzatti B., Bidoli E., Flaiban C., Bomben F., Piccinin M., Gipponi K.M., Mariutti G., Busato S., Mella S. (2019). Hospital Anxiety and Depression Scale (HADS) accuracy in cancer patients. Support. Care Cancer.

[34] Iani L., Lauriola M., Costantini M. (2014). A confirmatory bifactor analysis of the Hospital Anxiety and Depression Scale in an Italian community sample. Health Qual. Life Outcomes.

[35] Qiu J., Shen B., Zhao M., Wang Z., Xie B., Xu Y. (2020). A nationwide survey of psychological distress among Chinese people in the COVID-19 epidemic: Implications and policy recommendations. Gen. Psychiatry.

[36] Costantini A., Mazzotti E. (2020). Italian validation of COVID-19 Peritraumatic Distress Index and preliminary data in a sample of general population. Riv. Psichiatr..

[37] Steyn H.S., Ellis S.M. (2009). Estimating an Effect Size in One-Way Multivariate Analysis of Variance (MANOVA). Multivar. Behav. Res..

[38] Faul F., Erdfelder E., Lang A.G., Buchner A. (2007). G*Power 3: A flexible statistical power analysis program for the social, behavioral, and biomedical sciences. Behav. Res. Methods.

[39] Muls A., Georgopoulou S., Hainsworth E., Hartley B., O’Gara G., Stapleton S., Cruickshank S. (2022). The psychosocial and emotional experiences of cancer patients during the COVID-19 pandemic: A systematic review. Seminars in Oncology.

[40] Ferrara M., Langiano E., Falese L., De Marco A., De Vito E. (2021). Quality of Life and Psychosocial Impacts of the Different Restrictive Measures during One Year into the COVID-19 Pandemic on Patients with Cancer in Italy: An Ecological Study. Int. J. Environ. Res. Public Health.

[41] Madkhali N.A., Ameri A., Al-Naamani Z.Y., Madkhali M.A., Alshammari B., MA A.L. (2022). Has the COVID-19 pandemic affected the psychological state of arab cancer patients?. Curr. Psychol..

[42] Bivia-Roig G., La Rosa V.L., Gomez-Tebar M., Serrano-Raya L., Amer-Cuenca J.J., Caruso S., Commodari E., Barrasa-Shaw A., Lison J.F. (2020). Analysis of the Impact of the Confinement Resulting from COVID-19 on the Lifestyle and Psychological Wellbeing of Spanish Pregnant Women: An Internet-Based Cross-Sectional Survey. Int. J. Environ. Res. Public Health.

[43] Commodari E., La Rosa V.L., Carnemolla G., Parisi J. (2021). The psychological impact of the lockdown on Italian university students during the first wave of COVID-19 pandemic: Psychological experiences, health risk perceptions, distance learning, and future perspectives. Mediterr. J. Clin. Psychol..

